# Effects of a new intervention based on the Health at Every Size approach for the management of obesity: The “Health and Wellness in Obesity” study

**DOI:** 10.1371/journal.pone.0198401

**Published:** 2018-07-06

**Authors:** Mariana Dimitrov Ulian, Ana Jéssica Pinto, Priscila de Morais Sato, Fabiana B. Benatti, Patricia Lopes de Campos-Ferraz, Desire Coelho, Odilon J. Roble, Fernanda Sabatini, Isabel Perez, Luiz Aburad, André Vessoni, Ramiro Fernandez Unsain, Marcelo Macedo Rogero, Tatiana Natasha Toporcov, Ana Lúcia de Sá-Pinto, Bruno Gualano, Fernanda B. Scagliusi

**Affiliations:** 1 Department of Nutrition, School of Public Health, University of Sao Paulo, Sao Paulo, Brazil; 2 Applied Physiology & Nutrition Research Group, Laboratory of Assessment and Conditioning in Rheumatology, Faculdade de Medicina FMUSP, Universidade de Sao Paulo, Sao Paulo, Brazil; 3 School of Applied Sciences, Universidade Estadual de Campinas, Limeira, Brazil; 4 Faculty of Physical Education, Universidade Estadual de Campinas, Campinas, Brazil; 5 Institute of Health and Society, Federal University of Sao Paulo, Sao Paulo, Brazil; 6 Food Research Center (FoRC), CEPID-FAPESP, Research Innovation and Dissemination Centers Sao Paulo Research Foundation, Sao Paulo, Brazil; 7 Department of Epidemiology, School of Public Health, University of Sao Paulo, Sao Paulo, Brazil; 8 Laboratory of Assessment and Conditioning in Rheumatology, Faculdade de Medicina FMUSP, Universidade de Sao Paulo, Sao Paulo, Brazil; University of Newcastle, AUSTRALIA

## Abstract

Health at Every Size^®^ (HAES^®^) is a weight-neutral approach focused on promoting healthy behaviors in people with different body sizes. This study examined multiple physiological, attitudinal, nutritional, and behavioral effects of a newly developed, intensive, interdisciplinary HAES^®^-based intervention in obese women. This was a prospective, seven-month, randomized (2:1), controlled, mixed-method clinical trial. The intervention group (I-HAES^®^; n = 39) took part in an intensified HAES^**®**^-based intervention comprising a physical activity program, nutrition counseling sessions, and philosophical workshops. The control group (CTRL; n = 19) underwent a traditional HAES^**®**^-based intervention. Before and after the interventions, participants were assessed for physiological, psychological, and behavioral parameters (quantitative data) and took part in focus groups (qualitative data). Body weight, body mass index, and waist and hip circumferences did not significantly differ within or between groups (P > 0.05). I-HAES^®^ showed increased peak oxygen uptake and improved performance in the timed-stand test (P = 0.004 and P = 0.004, between-group comparisons). No significant within- or between-group differences were observed for objectively measured physical activity levels, even though the majority of the I-HAES^®^ participants indicated that they were engaged in or had plans to include physical activity in their routines. I-HAES^®^ resulted in improvements in eating attitudes and practices. The I-HAES^®^ group showed significantly improved all Body Attitude Questionnaire subscale and all Figure Rating Scale scores (P ≤ 0.05 for all parameters, within-group comparisons), whereas the CTRL group showed slight or no changes. Both groups had significant improvements in health-related quality of life parameters, although the I-HAES^®^ group had superior gains in the “physical health,” “psychological health,” and “overall perception of quality of life and health” (P = 0.05, 0.03, and 0.02, respectively, between-group comparisons) domains. Finally, most of the quantitative improvements were explained by qualitative data. Our results show that this new intensified HAES^®^-based intervention improved participants’ eating attitudes and practices, perception of body image, physical capacity, and health-related quality of life despite the lack of changes in body weight and physical activity levels, showing that our novel approach was superior to a traditional HAES^®^-based program.

## Introduction

The rising rates of obesity and the consequent health-related effects and healthcare costs have highlighted the need for approaches to assist people who are obese. The majority of obesity interventions focus on weight loss through a restrictive diet and physical activity programs. However, the negative effects provoked by interventions of this nature include binge eating and eating disorders, body dissatisfaction, low self-esteem, and culpability and stigmatization of the fat body [1,2,3]. Moreover, the success rate of weight-loss approaches regarding sustainable weight loss, reduction of body fat mass, maintenance of body fat-free mass, and other health benefits (e.g., clinical improvement in blood pressure, lipid profile, physical activity levels, disturbed eating behaviors, self-esteem, and body image) [4,5] is limited. In this context, weight-neutral approaches, such as the Health at Every Size^®^ approach (HAES^®^), are of increasing interest [6] (Health at Every Size^®^ and HAES^®^ are registered trademarks of the Association for Size Diversity and Health—ASDAH).

HAES^®^ aims to promote healthy behaviors in people with different body sizes, regardless of body weight changes. Overall, the approach encourages the development of a positive body image and the acceptance of different body shapes and sizes; the promotion of eating practices that respect individual nutritional needs and the sensations of hunger, satiety, appetite, and pleasure; the promotion of enjoyable and sustainable physical activities [7].

HAES^®^-based interventions have been shown to improve participant’s diet, eating patterns, eating behaviors, anthropometric and metabolic parameters, and psychological well-being [8–15]. However, HAES^®^-based interventions are traditionally characterized by group meetings and fixed discussion topics conducted by a limited number of professionals and do not effectively promote physical activity or assess it as an outcome. Moreover, qualitative evaluation is rarely performed, precluding more comprehensive conclusions regarding participants’ experiences and opinions [8,9,11–17].

In a pilot study, we showed that a 1-year HAES^®^-based intervention comprising an exercise program, nutrition counseling, and philosophical workshops led to improvements in body composition, body dissatisfaction, perception of body size, and symptoms of binge eating in addition to participants reporting behavioral and attitudinal changes towards eating and physical activity, as well as improved food choices [18,19]. However, the study had a quasi-experimental design, a small sample size, and did not evaluate cardiovascular risk factors. Given the clear potential of non-prescriptive interdisciplinary interventions for the management of obesity, this study aimed to use a mixed-method approach and a randomized controlled design to extensively investigate the effects of an intensive interdisciplinary HAES^®^-based intervention on multiple physiological, attitudinal, nutritional, and behavioral parameters in obese women. Our hypothesis was that the new HAES^®^-based intervention would promote greater improvements in overall health-related outcomes (including weight loss, despite the weight-neutral nature of the programs) in comparison to a traditional HAES^®^-based intervention.

## Methods

### Study design and participants

The rationale and design of this study have been fully described elsewhere [20]. In brief, this was a prospective, seven-month, randomized, controlled, mixed-methods clinical trial. The sample consisted of 58 women aged between 25 and 50 years with body mass indexes (BMIs) ranging between 30 and 39.9 kg/m^2^. The exclusion criteria included: a) diagnosis of diabetes mellitus, congestive heart failure, chronic renal disease, or hepatic steatosis; b) use of medications, such as appetite suppressants, thyroid hormones, diuretics or any other “anti-obesity” drug; c) currently engaged in nutritional counseling or in regular supervised physical activity programs elsewhere; e) currently pregnant or nursing. Participants were randomly allocated to the intensified HAES^®^-based intervention (I-HAES^®^) or the control (CTRL) groups in a 2:1 ratio using the Research Randomizer software.

The project was approved by the institutional Ethics Committee of the School of Public Health, University of Sao Paulo (protocol 1.738.855). The participants signed an informed consent and all procedures were in accordance with the Declaration of Helsinki as revised in 2008. This study is registered at clinicaltrials.gov (NCT02102061).

### I-HAES^®^-based intervention

The I-HAES^®^ group participated in a program comprising thrice-weekly physical activity sessions, bimonthly individual nutritional sessions, and five philosophical workshops over 7 months. All professionals involved in the intervention were trained in the HAES^®^ concepts.

The physical activity program was supervised by professionals who had a bachelor degree in Physical Education. The sessions lasted for one hour and comprised different approaches aimed at increasing enjoyment and autonomy for engaging in daily physical activities (e.g., playing ludic games, dancing, engaging in different sports, exercising at participant’s preferred intensities). Improvements in physical capacity and weight loss were considered consequences of this process. As this is the first study to use the HAES^®^ principles to design physical activity sessions, our theoretical foundation is fully presented in data in S1 File.

The nutritional intervention was based on nutritional counseling [21], with no prescription of diets. We aimed to promote healthy eating habits by stimulating participants’ sensitivity to hunger and satiety cues, emotional eating, triggers that could lead to automatic behaviors related to food, and methods to neutralize food (i.e., to not classify food as “good” or “bad”). To do so, we used the following strategies: maintaining a food diary, meal planning, and goal setting in accordance with the HAES^®^ principles [20]. Before beginning the nutritional counseling, two 1-h lectures were held to present the nutritional approach and to address sociocultural aspects involving eating and concepts about healthy eating according to the Dietary Guidelines for the Brazilian Population [22]. Finally, participants received a book [23] that addressed the same principles encouraged by the nutritional intervention. The nutritional sessions lasted for 45 minutes and were conducted by dietitians, who had a bachelor degree in Nutrition. A full description of the nutritional intervention may be found elsewhere [18–20].

The philosophical workshops consisted of five 1-h meetings focused on discussion and reflection about topics related to obesity (e.g., concepts of desire and boredom [24], restriction and health [25,26], body and health moralization [27–29], freedom [24,30] and anxiety [31]. The philosophical workshops were conducted by a professional, who had a bachelor degree in Philosophy. The information regarding each component of this intervention is extensively described elsewhere [20].

### CTRL group

The CTRL group attended bimonthly educational lectures, in accordance to the HAES^®^ principles, on the same topics offered to the I-HAES^®^ group (i.e., healthful eating, physical activity and philosophy, and encouraging the adoption of a healthy lifestyle). This “control” intervention was designed to mimic the most traditional HAES^®^-based interventions [8,9–17], as described elsewhere [20]. Table 1 presents the type and frequency of the activities performed by each group.

**Table 1:** Type and frequency of the activities performed by the intensified HAES^®^-based intervention group (I-HAES^®^) and the control group (CTRL).

Activity	I-HAES^®^	CTRL
Supervised physical activity	3 times-a-week	none
Individual nutritional sessions	2 times per month	none
Philosophical workshops	5 throughout the intervention	none
Lectures on physical activity	none	1
Lectures on healthy eating	2 (pre-intervention)	2
Lectures on philosophy	none	1
Food diary register	Throughout the intervention	2 (pre- and post-intervention)

### Quantitative data collection and statistical analysis

As previously described [20], prior to and after the intervention (i.e., pre and post), all participants were assessed for anthropometry measures (body weight, height, and hip and waist circumferences); aerobic condition (using a maximal graded exercise test); spontaneous physical activity levels (objectively measured using an ActiGraph GT3X^®^ accelerometer [ActiGraph®, Pensacola, FL]); muscle function (using the timed-stands test [32] and timed-up-and-go test [33], where the lower the scores the better); psychological and behavioral assessments using validated questionnaires; namely, body perception and dissatisfaction (Figure Rating Scale [34,35]) attitudes towards the body (Body Attitude Questionnaire–BAQ–[36,37]) and health-related to quality of life (WHOQOL-BREF [38,39]). From the food diary data, we also calculated the frequency (considering total and daily intake) of intake of fruits, vegetables and ultra-processed foods, as a *post-hoc* analysis.

Mixed model analysis was performed for each dependent variable, with group (intervention and control groups) and time (pre and post) as fixed factors and subjects as a random factor. In the case of significant F-values, a *post hoc* test with Tukey’s adjustment was performed for multiple comparisons. For non-parametric data, independent and dependent samples were compared using Mann-Whitney U- and Wilcoxon tests, respectively. Percent delta changes were compared between groups [(post–pre from I-HAES^®^)–(post–pre from CTRL)] using unpaired Student’s *t*-tests. Participants’ baseline characteristics, and baseline characteristics between participants who retained and those who dropped out, were compared using Student’s *t*- or Chi-square tests. Finally, within-group Cohen’s d effect sizes (ES) were calculated [40]. All data analyses were performed using SAS 8.2 (SAS Institute Inc., Cary, NC, USA) or SPSS Statistics for Windows, version 17.0. The level of significance was set at P ≤ 0.05 and P values between 0.05 and 0.1 were considered to indicate a tendency towards significance. Quantitative data are presented as mean ± standard deviation or median ± interquartile range, estimated mean of differences between pre and post values, 95% confidence interval (CI), and % difference between delta change, except stated otherwise.

### Qualitative data collection and analysis

As described previously [20], two focus groups convened to understand participant experiences, feelings, expectations, and opinions regarding the intervention and the interdisciplinary team, aspects related to their eating practices, and quality of life. The I-HAES^®^ and CTRL groups attended separate focus groups. The initial focus groups, each composed of 7 to 10 participants, met one month after the beginning of the intervention and the final focus groups met in the last month of the intervention. Forty-eight and 14 participants in the I-HAES^®^ and CTRL groups, respectively, joined the initial focus groups whereas 32 and 11, respectively, joined the final focus groups.

For qualitative data, an exploratory content analysis was conducted using an inductive approach which revealed themes and codes from the data. Themes were selected by two researchers using the “cutting and sorting” approach and subsequently organized in a structured codebook [41]. Both coders independently applied the codebook to the data set, using phrases as the unit of analysis. Kappa coefficients for inter-rater reliability were calculated using the GraphPad QuickCalcs online software. Themes were analyzed considering their core and peripheral aspects, with attention to their details and co-occurrence [41]. The results of the exploratory content analysis are presented for each group with a detailed description, direct quotes, and paraphrases [41]. Speeches from the I-HAES^®^ group members are identified as “I” whereas those from the CTRL group are indicated as “C”.

## Results

### Participants

**Fig 1:**
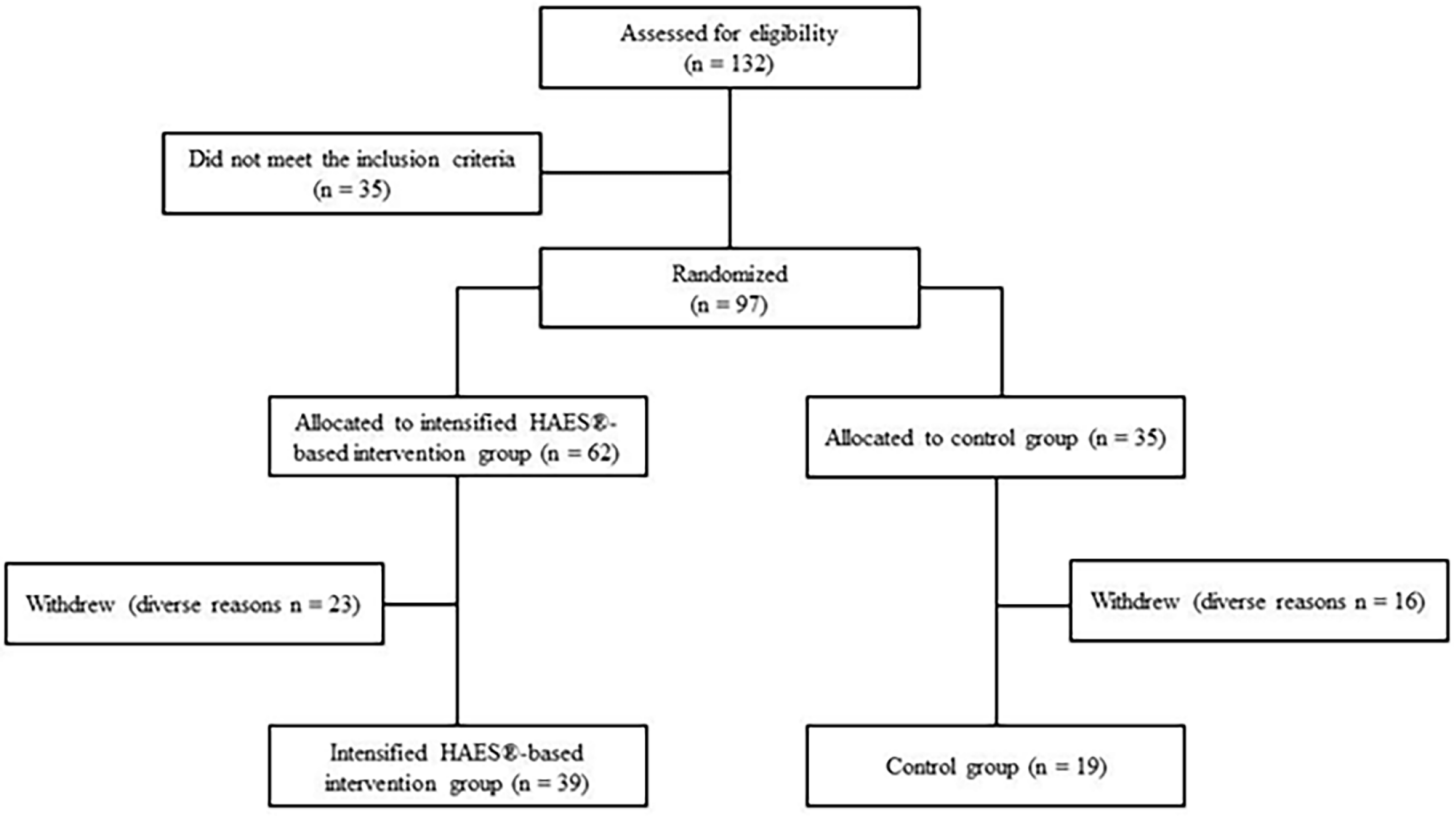
The “Health and Wellness in Obesity” study flowchart.

One hundred and thirty-two women were screened for participation. Ninety-seven met the inclusion criteria and were randomly assigned into either the I-HAES^®^ (n = 62) or CTRL (n = 35) groups. Thirty-nine participants withdrew from the study for various reasons (one moved to another country, two because of distance issues, three became pregnant, six because of personal reasons, eight because of health reasons, and 19 were not available). Therefore, 58 subjects completed the trial and were included in the analysis (I-HAES^®^, n = 39; CTRL, n = 19; maintaining the 2:1 ratio). S1 Table presents the main baseline characteristics between participants who retained and those who dropped out, stratified by groups. The study flowchart is presented in Fig 1.

Table 2 shows participant demographic characteristics at baseline. No differences were observed between groups at baseline regarding age, weight, BMI, waist and hip circumferences, education, and family income (Table 2).

**Table 2:** General characteristics of women participating in a randomized controlled trial based on the Health at Every Size^®^ approach.

	Intensified HAES^®^-based intervention group (n = 39)	Control group (n = 19)	*P*
**Age** (yr), mean, ± SD	33.4 ± 6.7	37.1 ± 7.8	0.065
**Anthropometry**			
Body weight (kg), mean, ± SD	90.7 ± 10.9	90.0 ± 10.5	0.912
Body mass index (kg/m^2^), mean, ± SD	34.5 ± 2.7	33.9 ± 3.1	0.552
Waist circumference (cm), mean, ± SD	108.6 ± 8.6	109.0 ± 10.2	0.248
Hip circumference (cm), mean, ± SD	119.4 ± 8.9	118.2 ± 7.0	0.431
**Relationship status**, n (%)			
Single	21 (54)	5 (26)	0.177
Married	13 (33)	10 (53)	
Common-law marriage	2 (5)	1 (5)	
Divorced	3 (8)	3 (16)	
**Education**, n (%)			
Graduated from high school	6 (15)	1 (5)	0.292
Incomplete high school graduation	0 (0)	0 (0)	
Graduated from college	18 (46)	7 (37)	
Incomplete college graduation	7 (18)	2 (11)	
Postgraduate-level studies	8 (21)	9 (47)	
**Monthly family income (value in Dollars)**, n (%)			
≤ 541.0	5 (13)	1 (5)	0.502
541.01–1,143.0	6 (15)	1 (5)	
1,143.01–2,705.0	17 (44)	11 (58)	
2,705.01–5,410.0	9 (23)	6 (32)	
≥ 5,410.01	1 (3)	0 (0)	
Did not know	1 (3)	0 (0)	

Data are expressed as means ± SD, except when identified. No significant differences were found. (nonpaired *t*-test or chi-square test).

### Qualitative data analysis

**Fig 2:**
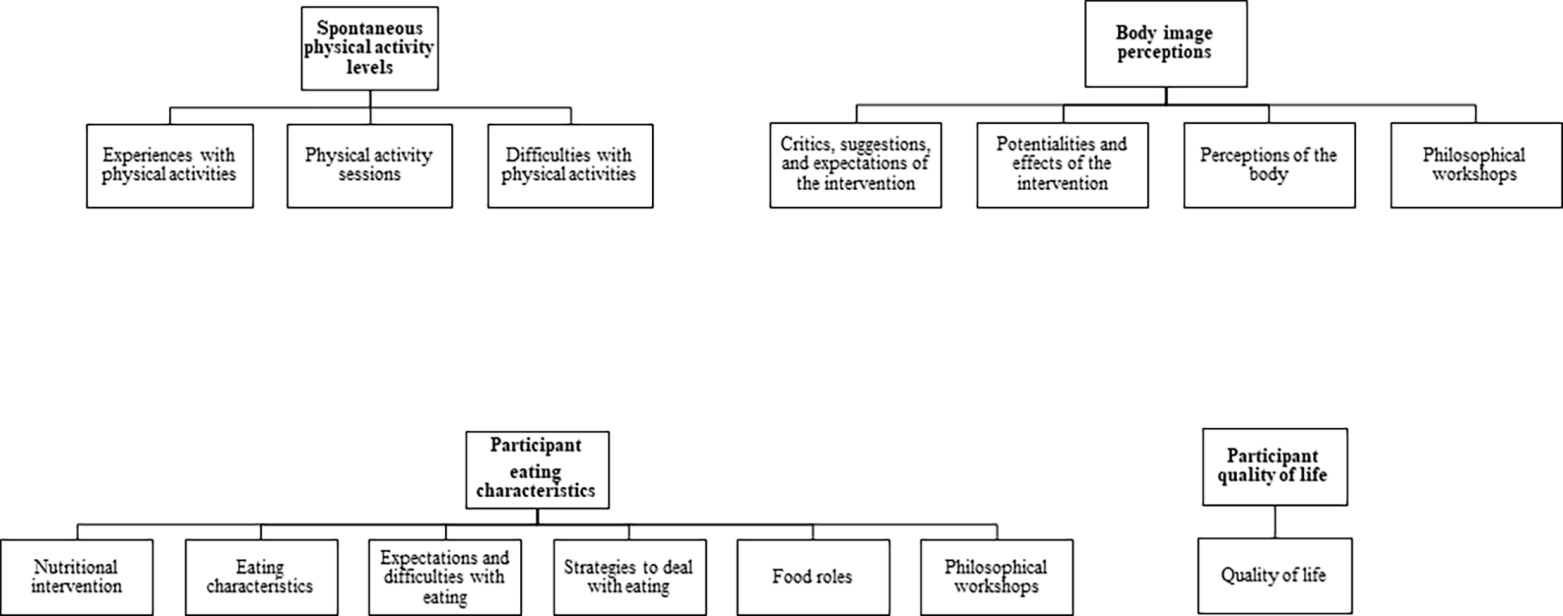
Representation of the codes that composed some of the resultant topics.

The thirteen emerging codes (Table 3) which resulted from participants’ statements revealed experiences that influenced their involvement with physical activity, eating-related aspects, body image, and quality of life. The kappa values showed a satisfactory strength of agreement between coders [42]. Codes that referred to similar topics were grouped and are shown in Fig 2. Notably, some of the resultant topics (spontaneous physical activity levels and participant eating, body image perceptions, and quality of life) were articulated by both quantitative and qualitative data, as presented in some of the following topics.

**Table 3:** Codes and kappa coefficients from focus groups with participants in the “Health and Wellness in Obesity” study.

Code	Kappa
Potentialities and effects of the intervention	0.854
Critics, suggestions, and expectations of the intervention	0.772
Quality of life	0.979
Perceptions of the body	0.662
Nutritional intervention	0.901
Eating characteristics	0.853
Expectations and difficulties with eating	0.738
Strategies to deal with eating	0.721
Food roles	0.652
Experiences with physical activities	0.608
Physical activity sessions	0.766
Difficulties with physical activities	0.978
Philosophical workshops	1.000

Kappa result is interpreted as follows: 0.61 to 0.80 as “good” agreement, and 0.81 to 1.00 as “very good” agreement [42].

### Anthropometry

Body weight, BMI, waist and hip circumferences, and waist to hip ratio did not significantly differ within or between groups (Table 4). Eight out of 39 participants from the I-HAES^®^ group and 1 out of 19 participants from the CTRL group achieved ≥ 5% weight loss, but this difference did not reach statistical significance (P = 0.246).

**Table 4:** Anthropometric, maximal aerobic capacity, muscle function, and physical activity assessments before and after the intervention.

	Intensified HAES^®^-based intervention group (n = 39)				Control group (n = 19)					
Variable	Pre	Post	CI (95%)	ES	Pre	Post	CI (95%)	ES	*% Difference between delta change*	*P value*
**Anthropometry**										
Body weight (kg)	90.7 (10.9)	90.1 (12.1)	-1.09 to 1.45	-0.06	90.0 (10.5)	91.0 (11.1)	-2.69 to 0.81	0.09	-0.87	0.301
Body mass index (kg/m^2^)	34.5 (2.7)	34.5 (3.2)	-0.42 to 0.56	0.01	33.9 (3.1)	34.3 (3.3)	-1.04 to 0.32	0.12	-0.83	0.309
Waist circumference (cm)	108.6 (8.6)	106.8 (8.1)	-0.66 to 4.19	-0.21	109.0 (10.2)	107.8 (9.6)	-2.18 to 4.54	-0.12	0.54	0.779
Hip circumference (cm)	119.4 (8.9)	119.0 (8.3)	-2.96 to 1.42	-0.04	118.2 (7.0)	118.3 (8.6)	-3.14 to 2.94	0.01	0.08	0.722
Waist to hip ratio	0.9 (0.06)	0.9 (0.06)	-0.006 to 0.031	-0.19	0.9 (0.07)	0.9 (0.08)	-0.017 to 0.034	-0.13	0.53	0.788
**Maximal aerobic capacity**										
Time-to-VAT (sec)	222.3 (70.1)	226.3 (56.1)	-30.35 to 22.45	0.06	243.3 (107.4)	203.4 (64.3)	2.67 to 79.73	-0.37	19.70^ǒ^	0.058
Time-to-RCP (sec)	454.7 (90.5)	502.6 (109.5)^#^	-86.96 to -9.10	0.53	463.3 (105.0)	446.6 (101.9)	-38.55 to 74.05	-0.16	15.02^ǒ^	0.059
Time-to-exhaustion (sec)*	625.4 (124.4)	671.4 (98.6)	-79.19 to -12.76	0.37	656.7 (91.4)	678.7 (108.0)	-71.18 to 26.01	0.24	3.84	0.429
VO_2_ VAT (mL/kg/min)	11.9 (3.0)	12.8 (2.7)	-1.82 to 0.02	0.30	12.2 (4.1)	11.2 (2.1)	-0.18 to 2.51	-0.26	17.83^ǒ^	0.014
VO_2_ RCP (mL/kg/min)	18.4 (2.9)	19.6 (3.6)^#^	-2.39 to -0.19	0.44	18.6 (3.6)	17.3 (2.4)^#^	-0.29 to 2.89	-0.34	14.65^ǒ^	0.010
VO_2peak_ (mL/kg/min)*	22.0 (3.8)	24.0 (3.9)^ø^	-2.89 to -1.07	0.52	22.5 (3.7)	22.2 (3.8)	-0.93 to 1.74	-0.09	10.91^ǒ^	0.005
**Muscle function tests**										
Timed Stands test (sec)	19.6 (4.0)	18.1 (4.3)^#^	0.29 to 3.10	-0.37	18.5 (4.4)	20.3 (5.8)	-3.62 to 0.27	0.41	0.51^ǒ^	0.007
Timed-up-and-go test (sec)*ᶿ	6.0 (0.8)	6.4 (1.2)	-0.87 to 0.19	0.52	7.3 (3.4)	8.1 (5.6)	1.54 to -0.08	0.24	-5.58	0.300
**Physical activity levels**										
Sedentary time (min/day)	513.5 (88.1)	501.1 (101.3)	-17.74 to 50.68	-0.14	538.5 (102.0)	517.0 (138.9)	-45.23 to 55.89	-0.21	2.63	0.715
Light PA time (min/day)	325.0 (57.5)	325.0 (71.8)	-24.18 to 28.19	0.00	328.2 (93.4)	369.2 (92.8)	-74.82 to 2.46	0.44	-8.34	0.106
Total MVPA (min/day)	39.3 (20.7)	39.8 (20.7)	-6.97 to 5.71	0.02	37.3 (21.6)	34.3 (23.5)	-7.53 to 11.22	-0.14	5.11	0.662

Data are expressed as mean (SD), estimated mean of differences between pre and post values, 95% confidence interval (CI), Cohen’s d effect sizes (ES), and % delta differences [(post–pre from intervention)–(post–pre from control)]; expressed as mean ± SD). VAT, ventilatory anaerobic threshold; RCP, respiratory compensation point; VO, oxygen uptake; VO2peak, peak oxygen uptake; PA, physical activity; MVPA, moderate-to-vigorous physical activity.

P-values for group vs. time interaction.

ø Significant difference within group (P ≤ 0.05)

# Tendency for significance within group (P ≤ 0.10)

* Significant main effect of time (P ≤ 0.05)

ᶿ Significant main effect of group (P ≤ 0.05)

ǒ Significant difference between delta changes (P ≤ 0.05)

### Aerobic conditioning

Table 4 shows data regarding aerobic capacity. There was a tendency towards an interaction effect in time-to-ventilatory anaerobic threshold (VAT) (P = 0.058) whereas delta analysis revealed a greater increase in time-to-VAT in the I-HAES^®^ group compared to that in the CTRL group (P = 0.04). An interaction effect was observed in VO_2_ at VAT (P = 0.01), but *post hoc* analyses did not detect any significant differences. However, delta analysis showed greater increases in VO_2_ at VAT in the I-HAES^®^ group compared to that in the CTRL group (P = 0.01).

A tendency towards an interaction effect was observed for time-to-respiratory compensation point (RCP) (P = 0.05), with *post hoc* analysis showing an increasing trend in the I-HAES^®^ group (P = 0.07); delta analysis revealed greater increases in the I-HAES^®^ group compared that in the CTRL group (P = 0.05). In addition, an interaction effect was observed for VO_2_ at RCP (P = 0.009), with the I-HAES^®^ and CTRL groups increasing and decreasing tendencies, respectively (P = 0.09; P = 0.06), whereas delta analysis showed greater increases in the I-HAES^®^ group compared to that in the CTRL group (P = 0.008).

A significant main effect of time was observed for the increase in time-to-exhaustion (P = 0.02), but no interaction effect was noted. Likewise, no significant differences were evidenced through delta analysis (P = 0.44). Finally, a main effect of time and an interaction effect were observed for VO_2peak_ (P = 0.05 and 0.004, respectively), with the I-HAES^®^ group (P = 0.0003), but not the CTRL group (P > 0.05), showing an increase in this parameter. Furthermore, delta analysis showed greater increases in VO_2peak_ in the I-HAES^®^ group compared to that in the CTRL group (P = 0.004).

### Muscle function

An interaction effect was observed in the timed-stand test (P = 0.006), with the I-HAES^®^ (P = 0.08), but not with the CTRL (P > 0.05) group showing an improving trend in this parameter. Similarly, delta analysis showed greater improvements in the timed-stand test in the I-HAES^®^ group compared to that in the CTRL group (P = 0.004). Significant main effects of group and time were observed for the timed-up-and-go test (P = 0.04 and 0.01, respectively), but no interaction effect was noted. No significant differences were evidenced through the delta analysis (P = 0.29) (Table 4).

### Physical activity levels

In the final focus groups, the majority of the participants in the I-HAES^®^ group reported being engaged in some physical activity outside the intervention context or having plans to include novel activities in their routines, suggesting that they had gained a willingness and autonomy to practice physical activities. The participants in the CTRL group reported that the lectures on physical activities had stimulated them to become more attentive about how much they moved their bodies and were more aware of its importance. In the final focus group, ten CTRL participants reported having included physical activities in their routines. The participants that did not engage in any physical activity reported that they could not identify which activity they enjoyed and mentioned aspects of their routines that acted as barriers (e.g., unforeseen events in their routines, work demands, caring for their children).

Accelerometry data did not reveal any changes within or between groups in sedentary time, light physical activity levels, or time spent in moderate to vigorous physical activity after the intervention. Likewise, the delta differences showed no significant differences in these parameters (P > 0.05) (Table 4).

### Participant eating characteristics

The I-HAES^®^ group showed significant decreases in the total and daily consumption of ultra-processed foods, and significant increases in the total and daily consumption of fruits and vegetables. The CTRL group did not show any difference in total and daily intake of abovementioned food groups (Table 5).

**Table 5:** Dietary frequency intake of ultra-processed foods, fruits and vegetables of women participating in a randomized controlled trial based on the Health at Every Size^®^ approach.

	Intensified HAES^®^-based intervention group (n = 39)			Control group (n = 19)		
	Pre	Post	*P*	Pre	Post	*P*
Ultra-processed foods (total consumption*), mean, ± SD	32.0 ± 17.0	21.7 ± 13.4	0.0001	28.3 ± 14.5	26.5 ± 14.8	0.363
Ultra-processed foods (daily consumption), mean, ± SD	4.6 ± 2.4	3.1 ± 1.9	0.0001	4.2 ± 2.0	3.8 ± 2.1	0.133
Fruits (total consumption), mean, ± SD	8.8 ± 6.7	12.0 ± 8.9	0.026	8.6 ± 4.4	9.4 ± 5.8	0.291
Fruits (daily consumption), mean, ± SD	1.3 ± 1.0	1.7 ± 1.3	0.038	1.3 ± 0.8	1.4 ± 0.8	0.333
Vegetables (total consumption), mean, ± SD	13.9 ± 9.4	18.9 ± 12.6	0.018	13.7 ± 9.7	13.5 ± 7.8	0.753
Vegetables (daily consumption), mean, ± SD	2.0 ± 1.3	2.7 ± 1.8	0.012	2.0 ± 1.4	1.9 ± 1.1	0.572

* At pre-intervention, food diaries were completed 6.9 ± 0.3 days and 6.6 ± 0.7 days in the intensified HAES^®^-based intervention group and the control group, respectively. At post-intervention, food diaries were completed 6.9 ± 0.5 days and 6.9 ± 0.5 days in the intensified HAES^®^-based intervention group and the control group, respectively. Data was analyzed by the Wilcoxon test.

The qualitative data corroborate these findings. In the I-HAES^®^ group, the initial eating difficulties were related to the eating structure, managing desires and emotional eating, eating in social situations, and knowing what and how much to eat. The participants had previously dealt with these issues by avoiding eating, replacing “high-calorie” foods with “low-calorie” foods, eating alone, and trusting other people to make their food choices. These participants completed food diaries throughout the intervention and reported that this tool led to the improvement of their eating perception and consciousness. This was particularly connected with improvements in sensitivity to hunger and satiety feelings, which allowed them to choose how much and what to eat. The participants were encouraged to increase their attentiveness while eating (i.e., eating without distractions, spending more time at the table, and evaluating what they felt like eating), resulting in changes in their food consumption: *“I’ve learned that after I stopped eating in front of the television I stopped nibbling”* (I10). Participants reported that they were able to identify when they relied on food to deal with feelings and emotions and were able to manage this situation differently. This gave them a sense of empowerment and responsibility for their own eating behaviors. The philosophical workshops were also reported to contribute to this change. In addition, members of the I-HAES^®^ group reported having a more diversified eating habit, increasing their consumption of *in natura* or minimally processed foods. Finally, they reported having acquired autonomy to plan their eating: they were able to anticipate and think about their eating, plan their grocery list, and organize their schedule to cook. Regarding cooking, they were stimulated to engage more often in this activity and to diversify the ingredients and spices they used, which resulted in a higher willingness and interest to eat fresh and home-made foods.

In the CTRL group, the initial eating difficulties were related to emotional eating, which they dealt with by avoiding eating or eating alone. The participants received lectures about healthful eating and completed food diaries at the beginning and after the intervention respectively. The participants reported benefits from these diaries: they were able to notice patterns in their eating habits (e.g., by evaluating the reasons why they ate, how they ate, how they felt while eating, identifying when they ate in response to emotions, and gauging their hunger and satiety) and were working to change them. The lectures on healthy eating were reported to be clarifying, helping them to reconsider what they understood as healthy food, to value the importance of planning their eating, and to be more attentive about the quantity and the quality of their eating. Nonetheless, according to their statements, the CTRL group was unable to make concrete changes in their eating habits.

### Body image perceptions

**Fig 3:**
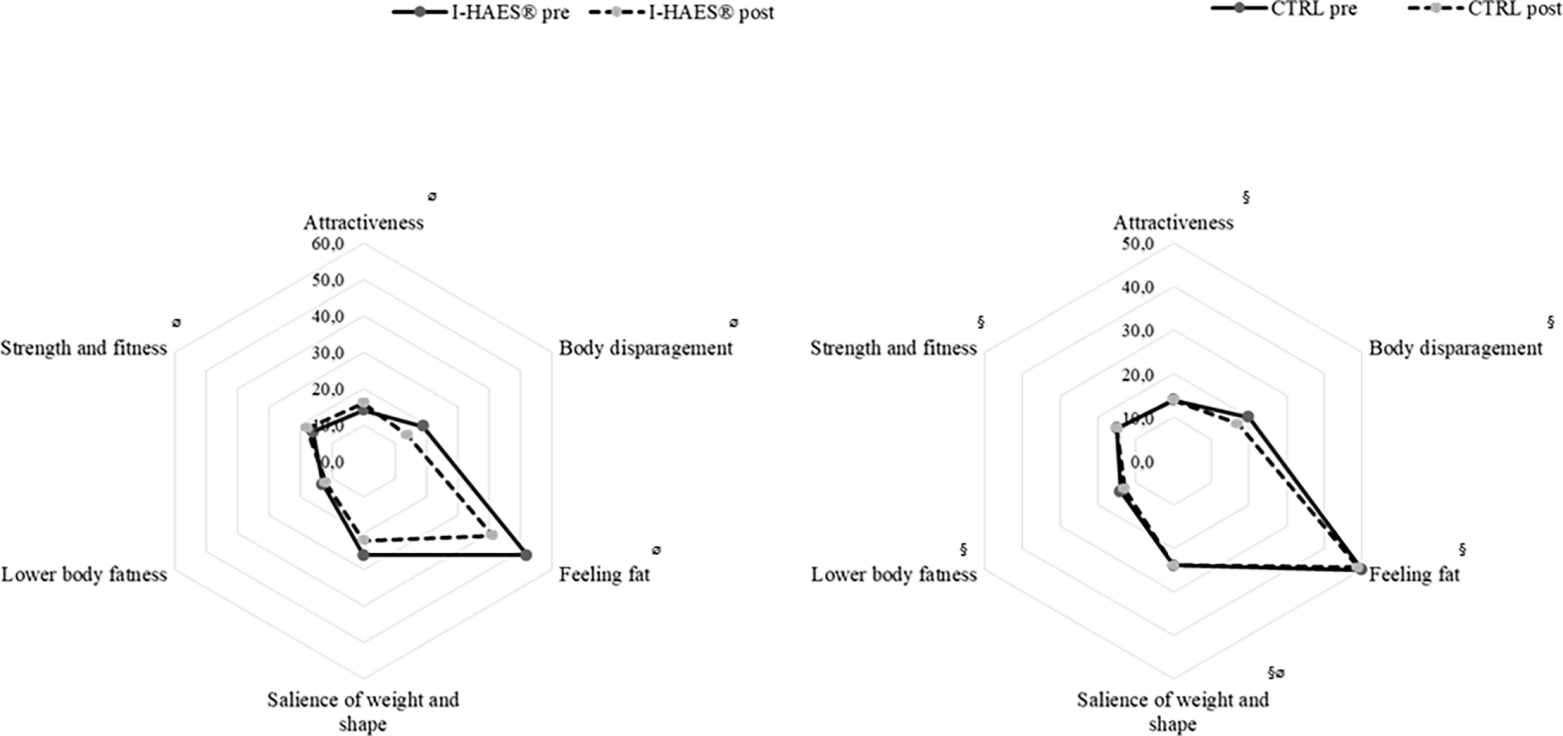
**(Left panel): Results from the intensified HAES**^**®**^**-based intervention group Body Attitude Questionnaire pre- and post-intervention. (Right panel): Results from the control group Body Attitude Questionnaire pre- and post-intervention.** I-HAES^**®**^, intensified HAES^®^-based intervention group; CTRL, control group. ^§^ Significant difference when compared to the intensified HAES^®^-based intervention group post-intervention (P ≤ 0.05). ^ø^ Significant difference within group (P ≤ 0.05).

Regarding participants’ attitudes toward their bodies, significant within-group differences were observed for all BAQ subscales post-intervention in the I-HAES^®^ group (P ≤ 0.05) (Fig 3). Compared to those in the CTRL group, the I-HAES^®^ group had significant decreases in the “body disparagement” (P = 0.01) and “feeling fat” (P = 0.01) subscale scores and a significant increase in the “attractiveness” (P = 0.01) and “strength and fitness” (P = 0.001) subscale scores post-intervention. In contrast, the CTRL group showed a decrease only in the “salience of weight and shape” subscale score (P = 0.03). In relation to participants’ body perception and dissatisfaction, the I-HAES^®^ group showed significant within-group differences for all Figure Rating Scale scores (P ≤ 0.05). The I-HAES^®^ group also showed improvements in the “current body size” and “current body size–ideal body size” scores when compared those in the CTRL group post-intervention (P = 0.02 for both parameters). In the “current body size” subscale, the I-HAES^®^ initial and final scores were 5.0 ± 3.0 and 2.0 ± 2.0, respectively (P = 0.001). The initial and final scores in the CTRL group were 3.0 ± 2.0 and 4.0 ± 5.0, respectively (P = 0.098). For “current body size–ideal body size”, the initial and final scores in the I-HAES^®^ group 3.0 ± 1.0 and 2.0 ± 2.0, respectively (P = 0.001) and 3.0 ± 2.0 and 3.0 ± 2.0, respectively in the CTRL group (P = 0.12).

The focus groups corroborated these quantitative changes. In the baseline focus group, both groups shared similar perceptions. While some participants reported having positive attitudes toward their bodies, valuing it as part of their personality, others did not: “*Losing weight is very important to me*: *I usually don’t look at myself in the mirror*, *and when I do I don’t recognize myself”* (I5), and *“I’d like to lose weight*, *it disturbs me*: *I stopped doing things*, *my social life changed”* (C47). Other participants shared a preoccupation with their health and willingness to perform activities or a desire to change certain characteristic of their bodies. In the final focus group, members of the I-HAES^®^ group said that they felt empowered by the activities, highlighting broader gains than nutrition, physical activity, and philosophy (*“The intervention changed my life*, *my conceptions*. *If I were to define the intervention as my life today*, *I would define it as life*, *death and rebirth*: *I lived*, *gained weight*, *died and the intervention rebirth me”–*I21). While four participants said that they still did not accept being fat, others said that although they expected to lose more weight, they were happy with their gains (e.g., more willingness, pain relief, etc.), and understood that body change would be a long-term process. Also, their weight was no longer a condition for their happiness (*“I used to see myself happy only after* [losing weight]. *I still want to lose weight*, *but I’m already happy now”*–I16). According to them, their previous focus on weight loss *per se* was a source of emotional distress that prevented them from keeping healthy habits as they were only sustained during weight loss. The philosophical workshop discussions were reported to have influenced these changes. These quantitative results suggest that, despite not having a significant weight loss, our participants developed a better body image and were more comfortable and less dissatisfied with their current physical condition. Those in the CTRL group stressed that the lectures had an effect on their concepts, making them reflect on the information that was communicated: they started to think and felt more responsible for their body condition and reported feeling more comfortable about it. Despite that, these participants mentioned discontentment: *“I have trouble when I’m getting dressed*, *I want to die because nothing fits me*, *everything looks hideous”* (C47).

### Participant quality of life

**Fig 4:**
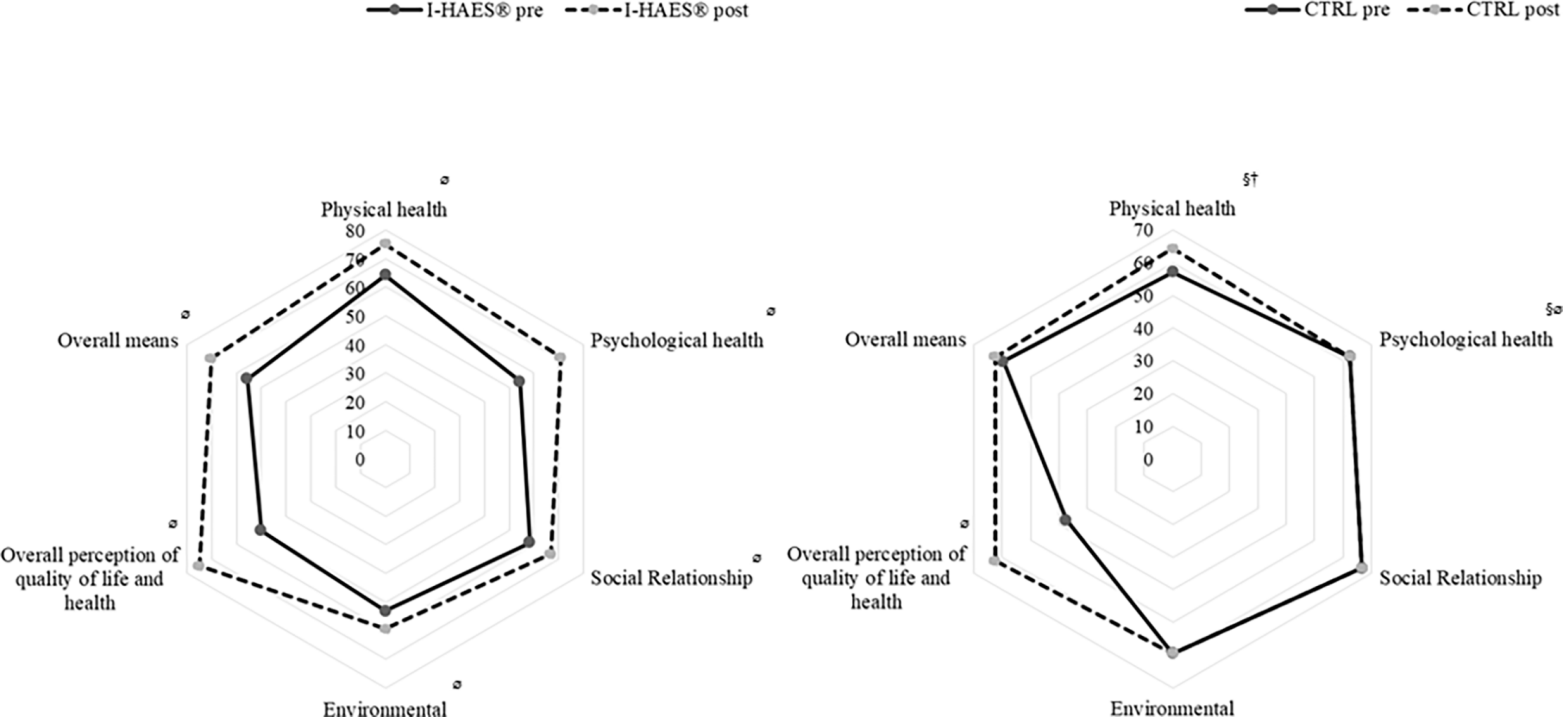
**(Left panel): Results from the intensified HAES**^**®**^**-based intervention group WHOQOL-BREF questionnaire pre- and post-intervention. (Right panel): Results from the control group WHOQOL-BREF questionnaire pre and post intervention.** I-HAES^®^, intensified HAES®-based intervention group; CTRL, control group. ^§^ Significant difference when compared to intensified HAES^®^-based intervention group post-intervention (P ≤ 0.05). ^ø^ Significant difference within group post-intervention (P ≤ 0.05). ^†^ Significant difference when compared to intensified HAES^**®**^-based intervention group pre-intervention (P ≤ 0.05).

In the I-HAES^®^ group, significant within-group differences were observed for all WHOQOL-BREF domains after the intervention (P ≤ 0.05) (Fig 4). Compared to those in the CTRL group, the I-HAES^®^ group showed significant improvements in the “physical health” (P = 0.05), “psychological health” (P = 0.02), and “overall perception of quality of life and health” (P = 0.03) domains. The CTRL group showed improvements only in the “psychological health” (P = 0.04) and “overall perception of quality of life and health” (P = 0.01) domains.

The focus groups shed light on the quantitative changes. Initially, in the I-HAES^®^ group, the quality of life was defined as a set of conditions that were not part of the participants’ routine and was related to expectations about their eating, physical activity, physical appearance, and well-being. In the final focus group, the intervention itself was part of the quality of life in the I-HAES^®^ group (*“quality of live is to come here and have fun doing the activities”–*I14). They reported having included aspects they considered important to their quality of life that were previously only hypothetical: “*I said before that quality of life would be to sleep better*, *eat well*, *have leisure time*. *Now I’m trying to sleep better*, *I’m hanging out more*” (I35). The CTRL group reported in both focus groups a general definition of quality of life and did not seem to have incorporated the aspects discussed in the intervention to improve their quality of life or to change their routines to approximate to what they considered to be quality of life.

## Discussion

The main finding of this study was that a new intensive, interdisciplinary HAES^®^-based, non-prescriptive intervention in obese women improved eating attitudes and practices, perception of body image, physical capacity, and health-related quality of life. Moreover, qualitative and quantitative data suggest that this novel intervention was more effective than a traditional HAES^®^-based one.

Throughout the seven-month follow-up period, both the I-HAES^®^ and CTRL groups showed no change (or slight reductions in the case of waist circumference) in anthropometric measurements. This find might be explained because the current physical activity program, tailored to meet the HAES^®^ principles, was not focused on exercise intensity or even on the adherence to the supervised exercise session; rather, the program was focused on promoting one’s self-engagement in playful, enjoyable activities, which may have been not sufficient to elicit detectable changes in anthropometric variables in such a follow-up period. However, this study was limited by the fact that body composition was not assessed, therefore we cannot rule out the possibility that participants did increase lean mass while decreasing body fat, without affecting body weight or other anthropometric parameters. In contrast, our I-HAES^®^ intervention improved aerobic conditioning and muscle function whereas no changes were observed in the CTRL group. Importantly, poor aerobic conditioning and function are associated with premature mortality in various populations [43,44]. In this context, Ross and Janiszewski [45] argue that anchoring the practice of exercise with weight reduction can mask opportunities to stimulate people to become more physically active [45]. Indeed, our data corroborate previous studies showing that regardless of body weight change, physical activity is associated with increased physical capacity [45–48], reinforcing the protective cardiometabolic effect of physical activity.

Participants in the I-HAES^®^ group were able to overcome their initial eating difficulties, reporting that they were able to better manage their emotional eating, eat in accordance with feelings of hunger and satiety, eat attentively, and improve the quality of food consumed. These gains are in accordance to those reported by previous traditional HAES^®^-based interventions [8,49,50]. In this study, we also showed that an intensive HAES^®^-based intervention led to improvements in participants’ autonomy to plan their eating and increased engagement with cooking, which might be important for maintenance of the habits acquired during the intervention. Notably, the CTRL group reported being stimulated to think about their eating, which did not provide them with autonomy to deal with their eating or result in sustainable changes. While traditional HAES^®^-based interventions have been shown in quantitative assessments to stimulate eating changes [9,12], no studies have qualitatively explored the participants’ likelihood to maintain these changes.

Our findings revealed that members of the I-HAES^®^ group developed a better perception of their bodies and were more comfortable and less dissatisfied with their current physical condition. Importantly, these changes were independent of any change in anthropometric measurements. In contrast, the CTRL group showed small or no changes in these perceptions. Altogether, these results suggest that the positive effects on participants’ body image observed in traditional HAES^®^-based interventions [16,17] were expanded by this novel intensive and interdisciplinary HAES^®^-based intervention, which may have provided an atmosphere of mutual empathy, encouraged self-worthiness, and promoted positive changes in participants’ body attitudes, perception, and dissatisfaction.

This intervention also improved the health-related quality of life, which might be a result of the abovementioned improvements. To our knowledge, this is the first intervention to use qualitative data to explore aspects of quality of life in a HAES^®^-based intervention. Brown et al. [51] explored obese patients’ experiences and perceptions of support in primary care and found that stigma-related thoughts were detrimental to participant quality of life and negatively affected interactions with health services. Our intervention was seemingly able to tackle numerous issues in a supportive way, helping to create a positive and welcoming environment for the participants and positively affecting their interactions with the intervention. The discussions promoted by the philosophical workshops were important for the promotion of reflection among our participants. These workshops are innovative in a HAES^®^-based intervention and should be considered in further studies. It is important to note that the CTRL group also showed improved “overall perception of quality of life and health”, suggesting that even small efforts regarding changes in health habits might improve the quality of life in this population.

Despite the aforementioned improvements, the accelerometry data did not show increases in physical activity levels in the I-HAES^®^ group. This was observed even though the participants reported having fun during physical activity classes and feeling empowered once they realized they could move their bodies in a number of ways; in addition, most reported engagement in some physical activity outside the intervention context or having plans to include novel activities in their routines. A possible explanation for this outcome is that structured exercise contributes only a minor daily physical activity energy expenditure, suggesting that exercise acts as a supplement to spontaneous physical activities [52]. Corroborating this notion, Turner et al. [53] provided healthy participants with significant amounts of prescribed exercise (almost 4 hours of exercise per week at 65% of their VO_2max_), which represented only 15% of the physical activity energy expenditure after 18 weeks of the intervention. Furthermore, there is data indicating that the adoption of structured exercise training may induce compensatory changes in behavior or physiology that could lead to decreased energy expenditure and spontaneous physical activity [54–61]. Therefore, it is necessary to test the efficacy and feasibility of programs focused primarily on the promotion of autonomy for physical activity and/or in reducing sedentary behavior in obese populations.

This study had some limitations. First, not all participants attended the focus groups (77% and 73% of adherence in the I-HAES^®^ and CTRL groups at pre-intervention, and 82% and 58% of adherence in the I-HAES^®^ and CTRL groups at post-intervention). However, the participants who attended the discussions expressed different ideas and, at some point, most of them were reinforced, indicating that the focus groups had reached saturation. Second, we did not evaluate body composition, which could have revealed a beneficial effect of exercise on lean and fat mass. Third, we were not able to evaluate participants who dropped out of the intervention and the relatively low retention may have contributed to some null findings. Finally, the major practical issue with lifestyle modifications lies in the long-term ability to sustain the short-term benefits. Although the current findings are promising, long-term studies should re-examine the feasibility, efficacy and effectiveness of this intervention.

## Conclusion

This novel seven-month intensive interdisciplinary HAES^®^-based non-prescriptive intervention in obese women improved participants’ eating attitudes and practices, perception of body image, physical capacity, and health-related quality of life despite no changes in body weight and spontaneous physical activity levels. Importantly, these results were corroborated through qualitative analysis showing that our novel approach was superior to that of a traditional HAES^®^-based program.

## Supporting information

S1 CONSORT Checklist (DOC)

S1 File A physical activity program based on the Health at Every Size^®^ approach–an innovative proposal. (DOCX)

S1 Table Baseline characteristics between participants who retained and those who dropped out, stratified by groups. Data are expressed as mean ± SD or n (%) of total sample per group. Significance level defined as p ≤ 0.05 (nonpaired *t*-test or chi-square test).
(DOCX)

## References

[1] Gormally J, Black S, Daston S, Rardin D. The assessment of binge eating severity among obese persons. Addict Behav. 1982;7(1):47–55. doi: 10.1016/0306-4603(82)90024-7 7080884 10.1016/0306-4603(82)90024-7

[2] ten Have M, de Beaufort I, Teixeira P, Mackenbach J, van der Heide A. Ethics and prevention of overweight and obesity: an inventory. Obes Rev. 2011; 12: 669–679. doi: 10.1111/j.1467-789X.2011.00880.x 21545391 10.1111/j.1467-789X.2011.00880.x

[3] Leske S, Strodl E, Hou X. A qualitative study of the determinants of dieting and non-dieting approaches in overweight/obese Australian adults. BMC Public Health. 2012;12(1). doi: 10.1186/1471-2458-12-1086 23249115 10.1186/1471-2458-12-1086 PMC3541951

[4] Tsai A, Wadden T. Systematic Review: An Evaluation of Major Commercial Weight Loss Programs in the United States. ACC Current Journal Review. 2005;14(5):15 doi: 10.1016/j.accreview.2005.04.012 10.7326/0003-4819-142-1-200501040-00012 15630109

[5] Aphramor L. Validity of claims made in weight management research: a narrative review of dietetic articles. Nutr J. 2010;9(1). doi: 10.1186/1475-2891-9-30 20646282 10.1186/1475-2891-9-30 PMC2916886

[6] Fulwiler C, Siegel JA, Allison J, Rosal MC, Brewer J, King JA. Keeping Weight Off: study protocol of an RCT to investigate brain changes associated with mindfulness-based stress reduction. BMJ Open. 2016; 6(11):e012573 doi: 10.1136/bmjopen-2016-012573 27903561 10.1136/bmjopen-2016-012573 PMC5168503

[7] (ASDAH) Association for Size Diversity and Health. Available at: https://www.sizediversityandhealth.org/. (2006)

[8] Carbonneau E, Bégin C, Lemieux S, Mongeau L, Paquette M, Turcotte M et al A Health at Every Size intervention improves intuitive eating and diet quality in Canadian women. Clin Nutr. 2017;36(3):747–754. doi: 10.1016/j.clnu.2016.06.008 27378611 10.1016/j.clnu.2016.06.008

[9] Mensinger J, Calogero R, Stranges S, Tylka T. A weight-neutral versus weight-loss approach for health promotion in women with high BMI: A randomized-controlled trial. Appetite. 2016;105:364–374. doi: 10.1016/j.appet.2016.06.006 27289009 10.1016/j.appet.2016.06.006

[10] Mensinger J, Calogero R, Tylka T. Internalized weight stigma moderates eating behavior outcomes in women with high BMI participating in a healthy living program. Appetite. 2016a;102:32–43. doi: 10.1016/j.appet.2016.01.033 26829370 10.1016/j.appet.2016.01.033

[11] Borkoles E, Carroll S, Clough P, Polman R. Effect of a non-dieting lifestyle randomised control trial on psychological well-being and weight management in morbidly obese pre-menopausal women. Maturitas. 2016;83:51–58. doi: 10.1016/j.maturitas.2015.09.010 26602363 10.1016/j.maturitas.2015.09.010

[12] Leblanc V, Provencher V, Bégin C, Corneau L, Tremblay A, Lemieux S. Impact of a Health-At-Every-Size intervention on changes in dietary intakes and eating patterns in premenopausal overweight women: Results of a randomized trial. Clin Nutr. 2012;31(4):481–488. doi: 10.1016/j.clnu.2011.12.013 22296874 10.1016/j.clnu.2011.12.013

[13] Provencher V, Bégin C, Tremblay A, Mongeau L, Corneau L, Dodin S et al Health-At-Every-Size and Eating Behaviors: 1-Year Follow-Up Results of a Size Acceptance Intervention. J Am Diet Assoc. 2009;109(11):1854–1861. doi: 10.1016/j.jada.2009.08.017 19857626 10.1016/j.jada.2009.08.017

[14] Provencher V, Bégin C, Tremblay A, Mongeau L, Boivin S, Lemieux S. Short-Term Effects of a “Health-At-Every-Size” Approach on Eating Behaviors and Appetite Ratings*. Obesity. 2007;15(4):957–966. doi: 10.1038/oby.2007.638 17426331 10.1038/oby.2007.638

[15] Carroll S, Borkoles E, Polman R. Short-term effects of a non-dieting lifestyle intervention program on weight management, fitness, metabolic risk, and psychological well-being in obese premenopausal females with the metabolic syndrome. Appl Physiol Nutr Metab. 2007;32(1):125–142. doi: 10.1139/h06-093 17332789 10.1139/h06-093

[16] Bacon L, Stern J, Van Loan M, Keim N. Size Acceptance and Intuitive Eating Improve Health for Obese, Female Chronic Dieters. J Am Diet Assoc. 2005;105(6):929–936. doi: 10.1016/j.jada.2005.03.011 15942543 10.1016/j.jada.2005.03.011

[17] Gagnon-Girouard M, Bégin C, Provencher V, Tremblay A, Mongeau L, Boivin S et al Psychological Impact of a “Health-at-Every-Size” Intervention on Weight-Preoccupied Overweight/Obese Women. J Obes. 2010;2010:1–12. doi: 10.1155/2010/928097 20798861 10.1155/2010/928097 PMC2925467

[18] Ulian MD, Benatti F, de Campos-Ferraz P, Roble O, Unsain R, de Morais Sato P et al The Effects of a “Health at Every Size®”-Based Approach in Obese Women: A Pilot-Trial of the “Health and Wellness in Obesity” Study. Front Nutr. 2015a; doi: 10.3389/fnut.2015.00034 26579524 10.3389/fnut.2015.00034 PMC4621435

[19] Ulian M, Gualano B, Benatti F, de Campos-Ferraz P, Roble O, Modesto B et al “Now I Can Do Better”: A Study of Obese Women's Experiences following a Nonprescriptive Nutritional Intervention. Clin Med Insights Womens Health. 2015b;8:CMWH.S23163 doi: 10.4137/CMWH.S23163 26417206 10.4137/CMWH.S23163 PMC4573064

[20] Ulian M, Gualano B, Benatti F, de Campos-Ferraz P, Coelho D, Roble O et al The design and rationale of an interdisciplinary, non-prescriptive, and Health at Every Size®-based clinical trial: The “Health and Wellness in Obesity” study. J Nutr Health. 2017;23(4):261–270. doi: 10.1177/0260106017731260 29214922 10.1177/0260106017731260

[21] Motta DG. Aconselhamento nutricional In: Motta DG. Educação nutricional & diabetes tipo 2. Piracicaba: Jacintha, 2009 pp. 27–33.

[22] Brasil. Ministério da Saúde (MS). Secretaria de Atenção à Saúde. Departamento de Atenção Básica. Guia Alimentar para a população brasileira. 2ed. Brasília: MS; 2014.

[23] Deram S. O peso das dietas. 1st ed. São Paulo: Sensus; 2014.

[24] Schopenhauer A. O mundo como vontade e representação. São Paulo: Unesp; 2005.

[25] Platão (2011) Fédon. Belém: EDUFPA.

[26] Espinosa B. Ética. Lisboa: Relógio D’água; 1992.

[27] Nietzsche F. Aurora. São Paulo: Companhia das Letras; 2004a.

[28] Nietzsche F. Genealogia da moral. São Paulo: Companhia das Letras; 2004b.

[29] Nietzsche F. Humano, demasiado humano. São Paulo: Companhia das Letras; 2004c.

[30] Sartre J-P. O ser e o nada. Petrópolis: Vozes; 2005.

[31] Kierkegaard S. The concept of anxiety. Princenton: Princenton University, 2013.

[32] Podsiadlo D, Richardson S. The Timed “Up & Go”: A Test of Basic Functional Mobility for Frail Elderly Persons. J Am Geriatr Soc. 1991;39(2):142–148. doi: 10.1111/j.1532-5415.1991.tb01616.x 1991946 10.1111/j.1532-5415.1991.tb01616.x

[33] Newcomer KL, Krug HE and Mahowald ML. Validity and reliability of the timed-stands test for patients with rheumatoid arthritis and other chronic diseases. J Rheumatol. 1993; 20:21–7. 8441160

[34] Stunkard AJ, Sorenson T, Schulsinger F. Use of Adaption Registry for the study of obesity and thinness In: The Genetics of Neurological and Pshychiatric Disorders. Raven Press, New York, 1983, pp. 115–120.

[35] Scagliusi F, Alvarenga M, Polacow V, Cordás T, de Oliveira Queiroz G, Coelho D et al Concurrent and discriminant validity of the Stunkard's figure rating scale adapted into Portuguese. Appetite. 2006;47(1):77–82. doi: 10.1016/j.appet.2006.02.010 16750589 10.1016/j.appet.2006.02.010

[36] Ben-Tovim D, Walker M. The development of the Ben-Tovim Walker Body Attitudes Questionnaire (BAQ), a new measure of women's attitudes towards their own bodies. ‎Psychol Med. 1991;21(03):775 doi: 10.1017/s0033291700022406 1946865 10.1017/s0033291700022406

[37] Scagliusi F, Polacow V, Coelho D, Philippi S, Cordás T, Alvarenga M et al Psychometric Testing and Applications of the Body Attitudes Questionnaire Translated into Portuguese. Percept Mot Skills. 2005;101(1):25–41. doi: 10.2466/pms.101.1.25-41 16350606 10.2466/pms.101.1.25-41

[38] WHOQOL Group (The). Development of the World Health Organization WHOQOL-BREF Quality of Life Assessment. Psychol Med. 1998;28(3):551–558. doi: 10.1017/s0033291798006667 9626712 10.1017/s0033291798006667

[39] Fleck MPA. O instrumento de avaliação de qualidade de vida da Organização Mundial da Saúde (WHOQOL-100): características e perspectivas. Cien Saude Colet. 2000; 5(1): 33–38.

[40] Cohen J. Statistical power analysis for the behavioral sciences. 2nd ed. Hillsdale, USA: Lawrence Erlbaum Associates; 1988

[41] Bernard R, Ryan GW. Analyzing Qualitative Data: Systematic Approaches. Thousand Oaks: Sage; 2010.

[42] Cohen J. A Coefficient of Agreement for Nominal Scales. ‎Educ Psychol Meas. 1960;20(1):37–46. doi: 10.1177/001316446002000104

[43] Lee D, Artero E, Sui Xuemei, Blair S. Review: Mortality trends in the general population: the importance of cardiorespiratory fitness. J Psychopharmacol. 2010;24(4_suppl):27–35. doi: 10.1177/1359786810382057 20923918 10.1177/1359786810382057 PMC2951585

[44] Blair S. Changes in physical fitness and all-cause mortality. A prospective study of healthy and unhealthy men. JAMA. 1995;273(14):1093–1098. doi: 10.1001/jama.273.14.1093 7707596

[45] Ross R, Janiszewski P. Is weight loss the optimal target for obesity-related cardiovascular disease risk reduction?. Can J Cardiol. 2008;24:25D–31D. doi: 10.1016/s0828-282x(08)71046-8 18787733 10.1016/s0828-282x(08)71046-8 PMC2794451

[46] Lee S. Exercise without weight loss is an effective strategy for obesity reduction in obese individuals with and without Type 2 diabetes. J Appl Physiol. 2005;99(3):1220–1225. doi: 10.1152/japplphysiol.00053.2005 15860689 10.1152/japplphysiol.00053.2005

[47] Dekker M, Lee S, Hudson R, Kilpatrick K, Graham T, Ross R et al An exercise intervention without weight loss decreases circulating interleukin-6 in lean and obese men with and without type 2 diabetes mellitus. Metabolism. 2007;56(3):332–338. doi: 10.1016/j.metabol.2006.10.015 17292721 10.1016/j.metabol.2006.10.015

[48] Slentz C, Duscha B, Johnson J, Ketchum K, Aiken L, Samsa G et al Effects of the Amount of Exercise on Body Weight, Body Composition, and Measures of Central Obesity. Arch Intern Med. 2004;164(1):31 doi: 10.1001/archinte.164.1.31 14718319 10.1001/archinte.164.1.31

[49] Schaefer J, Magnuson A. A Review of Interventions that Promote Eating by Internal Cues. J Acad Nutr Diet. 2014;114(5):734–760. doi: 10.1016/j.jand.2013.12.024 24631111 10.1016/j.jand.2013.12.024

[50] O'Reilly G, Cook L, Spruijt-Metz D, Black D. Mindfulness-based interventions for obesity-related eating behaviours: a literature review. Obes Rev. 2014;15(6):453–461. doi: 10.1111/obr.12156 24636206 10.1111/obr.12156 PMC4046117

[51] Brown I, Thompson J, Tod A, Jones G. Primary care support for tackling obesity: a qualitative study of the perceptions of obese patients. Br J Gen Pract. 2006; 56(530):666–672. 16953998 PMC1876632

[52] Thompson D. Physical activity for physiologists. Physiol News. 2013:32–5

[53] Turner J, Markovitch D, Betts J, Thompson D. Nonprescribed physical activity energy expenditure is maintained with structured exercise and implicates a compensatory increase in energy intake. Am J Clin Nut. 2010;92(5):1009–1016. doi: 10.3945/ajcn.2010.29471 20826629 10.3945/ajcn.2010.29471

[54] Di Blasio A, Ripari P, Bucci I, Di Donato F, Izzicupo P, D’Angelo E et al Walking training in postmenopause. Menopause. 2012;19(1):23–32. doi: 10.1097/gme.0b013e318223e6b3 21993080 10.1097/gme.0b013e318223e6b3

[55] Goran M, Poehlman E. Total energy expenditure and energy requirements in healthy elderly persons. Metabolism. 1992;41(7):744–753. doi: 10.1016/0026-0495(92)90315-2 1619993 10.1016/0026-0495(92)90315-2

[56] Manthou E, Gill JM, Wright A, Malkova D. Behavioural Compensatory Adjustments to Exercise Training In Overweight Women. Med Sci Sports Exerc. 2009;42(6):1121–8. doi: 10.1249/mss.0b013e3181c524b7 19997033 10.1249/MSS.0b013e3181c524b7

[57] Meijer E, Westerterp K, Verstappen F. Effect of exercise training on total daily physical activity in elderly humans. Eur J Appl Physiol Occup Physiol. 1999;80(1):16–21. doi: 10.1007/s004210050552 10367718 10.1007/s004210050552

[58] Meijer G, Janssen G, Westerterp K, Verhoeven F, Saris W, ten Hoor F. The effect of a 5-month endurance-training programme on physical activity: evidence for a sex-difference in the metabolic response to exercise. Eur J Appl Physiol Occup Physiol. 1991;62(1):11–17. doi: 10.1007/bf00635626 2007389 10.1007/BF00635626

[59] Morio B, Montaurier C, Pickering G, Ritz P, Fellmann N, Coudert J et al Effects of 14 weeks of progressive endurance training on energy expenditure in elderly people. Br J Nutr. 1998;80(06):511–519. doi: 10.1017/s0007114598001603 10211049 10.1017/s0007114598001603

[60] Westerterp K, Meijer G, Janssen E, Saris W, Hoor F. Long-term effect of physical activity on energy balance and body composition. Br J Nutr. 1992;68(01):21 doi: 10.1079/bjn19920063 1390606 10.1079/bjn19920063

[61] Melanson E, Keadle S, Donnelly J, Braun B, King N. Resistance to Exercise-Induced Weight Loss. Med Sci Sports Exerc. 2013;45(8):1600–1609. doi: 10.1249/MSS.0b013e31828ba942 23470300 10.1249/MSS.0b013e31828ba942 PMC3696411

