# Supplementary material for: Effects of a new intervention based on the Health at Every Size approach for the management of obesity: The “Health and Wellness in Obesity” study
Source: PLoS One. 2018 Jul 6;13(7):e0198401. doi: 10.1371/journal.pone.0198401 (PMC6034785; doi:10.1371/journal.pone.0198401)
Supplement: S1 File — (DOCX) [file pone.0198401.s002.docx]

**Supplementary file**

*A physical activity program based on the Health at Every Size^®^ approach – an innovative proposal*

The physical activity program constructs were based on the Health at Every Size^®^ (HAES^®^) principle of physical activity, which recommends the promotion of a pleasurable and sustainable physical activity practice [1]. Moreover, it also creates a positive environment to remove or minimize weight stigma [1]. Finally, other theoretical frameworks (i.e., experiences regarding exercise practice in Unified Healthcare System – SUS; i.e., Brazil’s publicly funded health care system) [2] and body practices as well as the Extended Clinic incorporated in the SUS [3,4] were included in the physical activity program’s constructs. Both frameworks recommend the promotion of pleasurable physical activity and also focus on participants’ needs, reinforce autonomy for physical activity practice, and encourage participants to actively engage in the planning of physical activity sessions.

The physical activity program was developed during transdisciplinary meetings including physical education professionals, nutritionists, and a philosopher. The main purpose of the physical activity program was to increase enjoyment and autonomy for engagement in daily physical activities in the most pleasant ways, in which improvements in “biological” parameters (e.g., aerobic capacity, muscle strength), development of motor skills, and weight loss were considered as a consequence of this process.

The physical activity program was held thrice-weekly at the University of Sao Paulo. It was not mandatory for participants and was supervised by physical education professionals trained in the intervention’s concepts. The physical activity sessions included a warm-up (approximately 10 minutes), main activity (approximately 45 minutes), and cool down (approximately 5 minutes). They created a positive environment for physical activity sessions, in which it was emphasized that physical activity should be pleasurable. Therefore, the participants were encouraged to perform the activities at a self-selected intensity and were free to perform another activity if they did not enjoy the proposed one (no participant asked to leave the sessions). The themes for each physical activity session were developed throughout the physical activity program according to physical education professionals’ perceptions and, mainly, on participants’ feedback, preferences and suggestions. A physical education professional observed each session and recorded field notes. Field notes are an instrument used to systematically register data or experiences to conduct a posteriori analysis. Throughout the intervention, two physical educators produced field notes in order to document their experiences, perceptions, and comments on the exercise sessions that could be relevant to the development and evaluation of the intervention. The notes were recorded during or after each exercise session and reported mainly perceptions about the feasibility and enjoyment of the activities proposed, participants’ feedback about the intervention or physical activity, and participants’ comments about physical activity during exercise sessions or outside the program. In general, the physical activity sessions involved discussions about the main recommendations regarding physical activity, promotion of different experiences to encourage its practice (e.g. sports, aerobic and strength conditioning, dance, childhood games, balance and flexibility exercises, etc.), and provided the required tools to undertake physical activity outside the intervention context. The main themes for the physical activity sessions are described below; the percentages indicate the extent of focus of each theme throughout the sessions:

1) Autonomy (13.2%): These sessions included activities designed to help participants to understand concepts related to physical activity (e.g., intensity, duration, frequency, and recovery); other activities were designed to provide tools for participants to engage in physical activity during free-living (e.g., examples of physical activities involving simple techniques and materials) and to discuss and propose strategies to overcome perceived barriers to physical activity (e.g., lack of time or place).

2) Physical capacities (23.7%): The main objective of these sessions was to explore different types of activities meant to increase one or more components of physical fitness (e.g., aerobic conditioning, muscle strength, flexibility, and balance). Moreover, the participants were provided with autonomy-related sessions. These were used as a basis for discussions regarding recommendations for physical capacity and related concepts, including intensity, frequency, and duration. Finally, the physical education professionals highlighted the importance of each specific physical capacity and encouraged the participants to identify their preferences regarding exercise and activities for each physical capacity.

3) Sports (36.8%): The participants were particularly interested in sports. The most suggested sports included volleyball, basketball, and soccer. The participants also showed an interest in sports that are not traditional in Brazil, such as rugby, badminton, and American football. Importantly, most of the sport-related activities were adapted for this program in order to improve the dynamic of the physical activities and motivate participants. Adaptations were made in order to make the activities possible in the intervention setting; for example, by mimicking each sport’s courts.

4) Traditional children's games (18.4%): These sessions involved mainly traditional children’s games, including dodgeball; relay races; and catch one, catch all. The participants were encouraged to share experiences and suggest games that they had played in childhood.

5) Dance (7.9%): Participants also suggested sessions involving different types of dance, although these were the minority of physical activity sessions.

The participants evaluated their experiences during the exercise sessions after the PA sessions, through an affectionateness Likert scale composed of five facial expressions ranging from sadness to happiness. Moreover, the participants were encouraged to write comments about their perceptions and feelings during the sessions. The affectionateness scale was completed at the end of each week based on the perceptions that had predominated during each exercise session. Finally, as mentioned above, the physical educators encouraged the participants to incorporate physical activity into their daily routines, counseling them during the exercise sessions and individually. Physical activity counseling was developed based on nutritional counseling, which was defined as “a meeting between two people to examine carefully, look with respect, and act with prudence and fairness on eating aspects of one of them” [5], and followed the guidelines of the Extended Nutritional Clinic [6]. In this sense, we defined physical activity counseling as a “meeting between two people to examine carefully, look with respect, and act with prudence and fairness on how to introduce physical activity in daily life considering feasibility, pleasure, and personal goals”. Therefore, in the middle of the physical activity program, the participants were invited to participate in individual physical activity counseling in order to encourage their engagement in physical activity during free-living. Physical activity counseling was held on a monthly basis by the physical education professionals. During these meetings, the participants were encouraged to share previous experiences and plans related to physical activity. The physical educators and participants jointly defined goals to accomplish these plans; these goals were designed to be progressive and easily achievable during the follow-up period (i.e., one month). Barriers and facilitators, autonomy, and myths related to physical activity experiences were also discussed. The follow-up meetings focused on accomplishing goals, discussing how to overcome barriers (if there was one during the follow-up), and setting new goals. Although all participants were invited to engage in this counseling, it was not obligatory and only 15 participants took part.

**References**

[1] (ASDAH) Association for Size Diversity and Health. Available at: https://www.sizediversityandhealth.org/. (2006)

[2] Florindo AA; Andrade DR. Experiências de Promoção da Atividade Física na Estratégia de Saúde da Família. 1. ed. Florianópolis: Sociedade Brasileira de Atividade Física e Saúde, 2015.

[3] Freitas FF, Carvalho YM, Mendes VM. Educação Física e Saúde: aproximações com a 'Clínica Ampliada'. Rev Bras Ciênc Esporte. 2013; 35:639-656.

[4] Mendes VM, Carvalho YM. Práticas Corporais & Clínica Ampliada. São Paulo: Hucitec, 2016. v. 1

[5] Motta DG. Aconselhamento nutricional. In: Motta DG. Educação nutricional & diabetes tipo 2. Piracicaba: Jacintha, 2009. pp. 27–33.

[6] Demétrio F, Paiva JB, Fróes AAG, Freitas MCS, Santos LAS. A nutrição clínica ampliada e a humanização da relação nutricionista-paciente: contribuições para reflexão. Rev Nutr. 2011; 24(5): 743-763.
