# Supplementary material for: Effects of a new intervention based on the Health at Every Size approach for the management of obesity: The “Health and Wellness in Obesity” study
Source: PLoS One. 2018 Jul 6;13(7):e0198401. doi: 10.1371/journal.pone.0198401 (PMC6034785; doi:10.1371/journal.pone.0198401)
Supplement: S1 Table — Data are expressed as mean ± SD or n (%) of total sample per group. Significance level defined as p ≤ 0.05 (nonpaired t-test or chi-square test). (DOCX) [file pone.0198401.s003.docx]

| **S1 Table. Baseline characteristics between participants who retained and those who dropped out, stratified by groups.** | | | | | | |
| --- | --- | --- | --- | --- | --- | --- |
|  | I-HAES^®^  (n = 39) | Drop outs  (n = 23) | *P value between I-HAES*^®^ *and drop outs* | CTRL  (n = 19) | Drop outs  (n = 16) | *P value between CTRL and drop outs* |
| **Age** (yr), mean, ± SD | 33.4 ± 6.7 | 36.1± 6.3 | 0.123 | 37.1 ± 7.8 | 35.1 ± 7.4 | 0.433 |
| **Anthropometry** |  |  |  |  |  |  |
| Body weight (kg), mean, ± SD | 90.7 ± 10.9 | 96.1 ± 9.8 | 0.057 | 90.0 ± 10.5 | 92.1 ± 13.9 | 0.623 |
| Body mass index (kg/m^2^), mean, ± SD | 34.5 ± 2.7 | 34.5 ± 8.0 | 0.981 | 33.9 ± 3.1 | 35.0 ± 2.5 | 0.254 |
| Waist circumference (cm), mean, ± SD | 108.6 ± 8.6 | 108.0 ± 9.8 | 0.798 | 109.0 ± 10.2 | 105.8 ± 12.8 | 0.492 |
| Hip circumference (cm), mean, ± SD | 119.4 ± 8.9 | 122.2 ± 7.0 | 0.222 | 118.2 ± 7.0 | 118.2 ± 9.0 | 0.981 |
| **Relationship status**, n (%) |  |  |  |  |  |  |
| Single | 21 (54) | 5 (22) | 0.032 | 5 (26) | 7 (44) | 0.611 |
| Married | 13 (33) | 16 (69) |  | 10 (53) | 7 (44) |  |
| Common-law marriage | 2 (5) | 0 (0) |  | 1 (5) | 0 (0) |  |
| Divorced | 3 (8) | 2 (9) |  | 3 (16) | 2 (13) |  |
| **Education**, n (%) |  |  |  |  |  |  |
| Graduated from high school | 6 (15) | 3 (13) | 0.053 | 1 (5) | 2 (13) | 0.019 |
| Incomplete high school graduation | 0 (0) | 1 (4) |  | 0 (0) | 1 (6) |  |
| Graduated from college | 18 (46) | 16 (70) |  | 7 (37) | 12 (75) |  |
| Incomplete college graduation | 7 (18) | 0 (0) |  | 2 (11) | 1 (6) |  |
| Postgraduate-level studies | 8 (21) | 3 (13) |  | 9 (47) | 0 (0) |  |
| **Monthly family income (value in Dollars)**, n (%) |  |  |  |  |  |  |
| ≤ 541.0 | 5 (13) | 1 (4) | 0.923 | 1 (5) | 1 (6) | 0.285 |
| 541.01 – 1,143.0 | 6 (15) | 7 (30) |  | 1 (5) | 3 (19) |  |
| 1,143.01 – 2,705.0 | 17 (44) | 7 (30) |  | 11 (58) | 1 (6) |  |
| 2,705.01 – 5,410.0 | 9 (23) | 4 (18) |  | 6 (32) | 2 (13) |  |
| ≥ 5,410.01 | 1 (3) | 0 (0) |  | 0 (0) | 0 (0) |  |
| Did not know | 1 (3) | 4 (18) |  | 0 (0) | 9 (56) |  |
| Data are expressed as mean ± SD or n (%) of total sample per group. Significance level defined as p ≤ 0.05 (nonpaired *t*-test or chi-square test). | | | | | | |
